# Transcriptional and Physiological Responses to Nutrient Loading on Toxin Formation and Photosynthesis in *Microcystis Aeruginosa* FACHB-905

**DOI:** 10.3390/toxins9050168

**Published:** 2017-05-17

**Authors:** Guotao Peng, Sijie Lin, Zhengqiu Fan, Xiangrong Wang

**Affiliations:** 1 Department of Environmental Science and Engineering, Fudan University, Shanghai 200433, China; zhqfan@fudan.edu.cn; 2 College of Environmental Science and Engineering, State Key Laboratory of Pollution Control and Resource Reuse, Tongji University, Shanghai 200092, China; lin.sijie@tongji.edu.cn

**Keywords:** microcystin, *mcy*, antioxidant system, nitrogen, phosphorus

## Abstract

An important goal of understanding harmful algae blooms is to determine how environmental factors affect the growth and toxin formation of toxin-producing species. In this study, we investigated the transcriptional responses of toxin formation gene (*mcyB*) and key photosynthesis genes (*psaB*, *psbD* and *rbcL)* of *Microcystis aeruginosa* FACHB-905 in different nutrient loading conditions using real-time reverse transcription quantitative polymerase chain reaction (RT-qPCR). Three physio-biochemical parameters (malondialdehyde (MDA), superoxide dismutase (SOD) and glutathione (GSH)) were also evaluated to provide insight into the physiological responses of *Microcystis* cells. We observed an upregulation of *mcyB* gene in nutrient-deficient conditions, especially in nitrogen (N) limitation condition, and the transcript abundance declined after the nutrient were resupplied. Differently, high transcription levels were seen in phosphorus (P) deficient treatments for key photosynthesis genes throughout the culture period, while those in N-deficient cells varied with time, suggesting an adaptive regulation of *Microsystis* cells to nutrient stress. Increased contents of antioxidant enzymes (SOD and GSH) were seen in both N and P-deficient conditions, suggesting the presence of excess amount of free radical generation caused by nutrient stress. The amount of SOD and GSH continued to increase even after the nutrient was reintroduced and a strong correlation was seen between the MDA and enzyme activities, indicating the robust effort of rebalancing the redox system in *Microcystis* cells. Based on these transcriptional and physiological responses of *M. aeruginosa* to nutrient loading, these results could provide more insight into *Microcystis* blooms management and toxin formation regulation.

## 1. Introduction

Blooms of toxic cyanobacteria have become a global environmental problem and a threat to the aquatic ecosystem and human health [1,2]. Among different algal species, *Microcystis* is of great interest because of the release of a potent hepatotoxin, microcystin (MC) [1,2,3,4]. The environmental factors, including temperature, light density, inorganic carbon and nutrient that drive *Microcystis* bloom formation, biological community structure and toxin production have been extensively studied. Some reports also demonstrated the changes of growth and MC production in *Microcystis aeruginosa* affected by other cyanotoxins, such as cylindrospermopsin [5,6]. Among the limiting factors, the important roles of nitrogen (N) and phosphorus (P), including nutrient availability and chemical forms, have gained widespread attention in recent years [7,8,9].

Since most freshwater ecosystems are P-limited, P loading was thought to act as a limiting factor in promoting the cyanobacterial blooms for decades [10,11]. However, conflicting reports exist. Some laboratory studies indicated that the P-deficient condition did not have impact on the growth rate of *Microcystis* [12,13], while others have showed that the growth of the same species declined in a P-limited condition primarily on account of a low carbon fixation rate [14]. Moreover, the effect of P availability on toxin production of *Microcystis* has been widely discussed, but still remains controversial [15,16]. Utkilen and Gjølme [17] stated that P-deficient conditions had no influence on the toxin production of *M. aeruginosa*, but iron-deficient conditions did. Others stated that increases of the MC concentration per dry weight were found under more P-limited conditions [14,18]. More recently, Kuniyoshi et al. [13] found that under a N/P ratio of 40:1 (P-deficient), the *M. aeruginosa* cells displayed high levels of *mcyD* transcript abundance along with intracellular MC content, which stressed the important role of N:P ratio in *mcy* transcription and MC production [15].

Besides P, N may be considered as having an equally important role in the occurrence of toxic, non-fixing cyanobacteria blooms, such as *Microcystis* [19,20]. Several laboratory studies have shown that increase of nitrate availability triggers *Microcystis* growth [21,22]. However, an excess of nitrate might inhibit the cells growth and reduce the photosynthetic activity consequently [8]. A binding site for the universal nitrogen regulator protein NtcA has been identified in the *mcy* bidirectional promotor region [23], which has sparked a debate on the subject of whether the MC production would be up- or down-regulated by the N availability. An increase of toxin amount was observed with increasing nitrate availability in *M. aeruginosa* [24,25], while opposite results have demonstrated an increase of MC production in N-limited conditions [26,27]. Sevilla et al. [21] reported that the MC concentration correlated with *mcyD* gene transcript, but both of them were independent from nitrate availability.

In either P or N limitation condition, reactive oxygen species (ROS) are generated due to the existence of several cross-regulatory reactions in cells, like nutrient acquisition, photosynthesis and redox control [28]. Under normal condition, concentration of ROS remains low due to the presence of antioxidant enzymes in cells, including catalase, superoxide dismutase, ascorbate peroxides, and glutathione [29]. The activities of these enzymes might be significantly enhanced due to an excess amount of ROS induced by stress [30,31], which reflect the rebalance of the physiological properties of *Microcystis* and help to understand the biochemical and molecular reactions in cells.

Numerous studies have been conducted to evaluate the effects of nutrient on the growth and biosynthesis of MC in *Microcystis* [15,26]. However, results are diversified and the cellular mechanism trigging the toxin production remains unclear. In light of the aforementioned conflicting reports, the aim of this study was to examine effects of nutrient loading on *M. aeruginosa* in terms of transcriptional and physiological responses. Target genes associated with toxin formation (*mcyB*) and photosynthesis (*psaB*, *psbD* and *rbcL*) were selected, along with three physio-biochemical parameters (malondialdehyde (MDA), superoxide dismutase (SOD) and glutathione (GSH)) to address this question. It is worth noting that we resupplied the nutrient to cells after nutrient starvation for several days, which has not been investigated previously and would provide insight to the recovery growth of *M. aeruginosa.* The results of this work could contribute to the existing literature by offering new insights into the ecological importance of nutrient for cyanobacteria cell metabolism.

## 2. Results

### 2.1. Cell Growth

**Figure 1:**
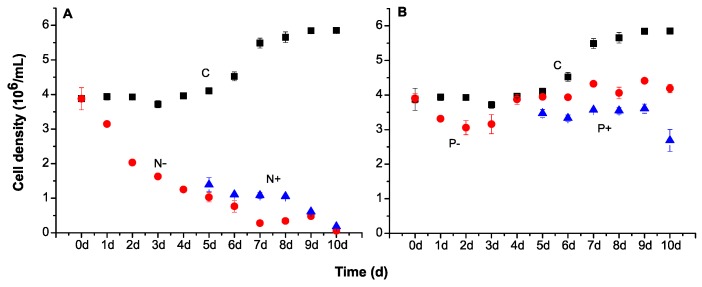
Cell density of *M. aeruginosa* at each treatment: nitrogen (**A**); and phosphorus (**B**). Different colors indicate different treatments: control (black), nutrient-deficient (red) and nutrient-added treatments (blue).

Cell abundance of *M. aeruginosa* was determined daily at each treatment (Figure 1). From the beginning of incubation, a significant decrease was seen in N-deficient treatments compared to control. After N was reintroduced on Day 5, the cell number turned to show a trend of increase but their growth was still significant lower than the control (Figure 1A). As for P treatments, a slight decrease was found in P-deficient group in the first three days but the cells presented a normal exponential growth from Day 4 (Figure 1B). No significant differences were seen between P-deficient cultures and the control on Day 5, although the maximum cell density was obtained in the control on Day 9. The cell density decreased after P was added on Day 5, but their number increased slightly later. Moreover, the number of cells under N treatments were significant lower than those of P treatments and control from Day 3, and the differences enhanced with the extension of the incubation time.

### 2.2. RT-qPCR Assay Performance

**Figure 2:**
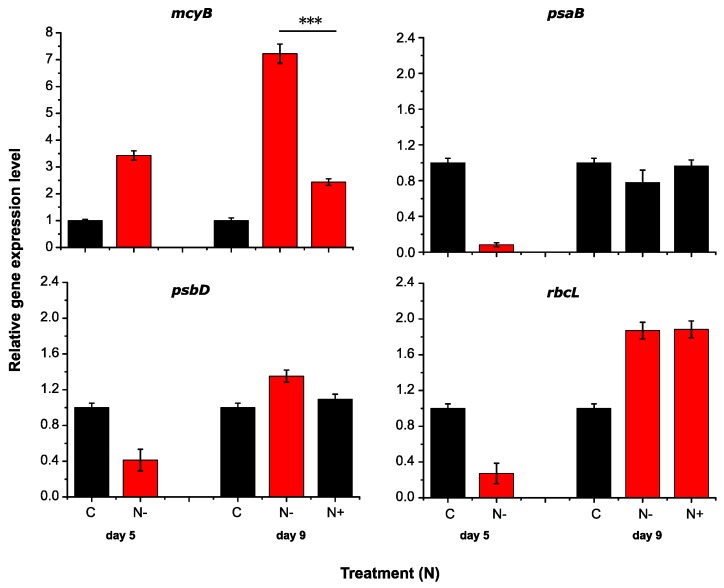
Relative transcription levels of target genes in N treatments. Red colors indicate the significant results compared to the control (*p* < 0.05). Significant differences are noted between N-deficient and N-added treatments (*p* < 0.001 ***, *p* < 0.01 **, *p* < 0.05 *).

**Figure 3:**
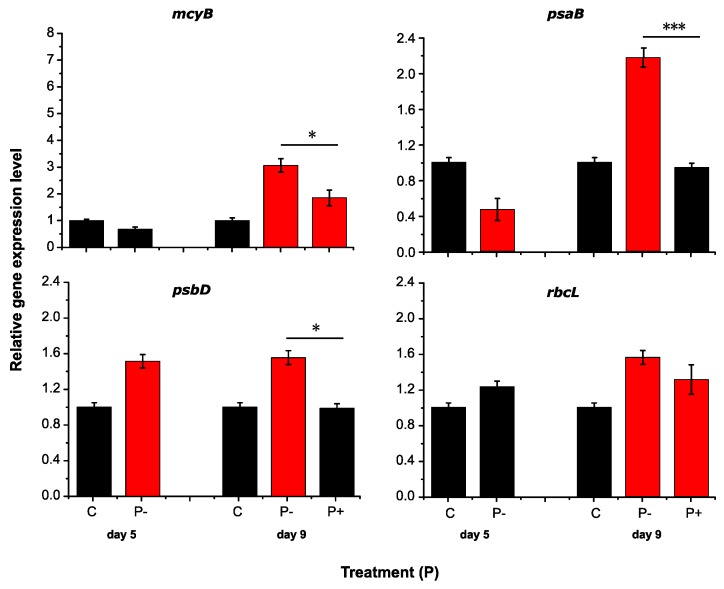
Relative transcription levels of target genes in P treatments. Red colors indicate the significant results compared to the control (*p* < 0.05). Significant differences are noted between P-deficient and P-added treatments (*p* < 0.001 ***, *p* < 0.01 **, *p* < 0.05 *).

Transcripts of key genes associated with MC formation (*mcyB*) and photosynthesis (*psaB, psbD, rbcL*) and were quantified via RT-qPCR (Figure 2 and Figure 3). Transcripts number of *mcyB* showed a significant increase in N-deficient treatments, about 3.5-fold higher than the control on Day 5, and the number increased markedly, reaching about 7.5-fold higher on Day 9. Interestingly, the expression of *mcyB* gene dropped 3.8-fold after N was added (Figure 2). In contrast, no significant differences were found between P-deficient treatments and the control on Day 5. The transcripts of P-deficient cultures showed a rising trend lately, but the differences narrowed after P was added (Figure 3). Compared to the nutrient-deficient groups, significant declines were seen when N or P was added, especially in N treatments (*p* < 0.001).

In comparison, transcriptional profiles for the key photosynthesis genes were quite different to the *mcyB* gene was seen. On Day 5, transcripts of all these three genes in N-deficient groups decreased significantly than the control. Different results were seen in P-deficient cultures. Apart from the significant decrease of *psaB* transcripts, the expression of the other two genes maintained at a high level (*rbcL*) or ever higher (*psbD,* about 1.5-fold) than the control (Figure 2 and Figure 3). More interestingly, the transcripts number increased dramatically in nutrient deficient groups and were significantly higher than the control on Day 9, except *psaB* gene in N-deficient cultures. After nutrient was supplied, no differences of *psaB* and *psbD* transcripts were seen in N and P-added treatments compared to the control, while the *rbcL* gene transcripts in those cultures were still higher. When comparing the nutrient-deficient and nutrient-added treatments, significant results were seen only in P treatments (*psaB* and *psbD*).

### 2.3. Examination of antioxidant systems

**Figure 4:**
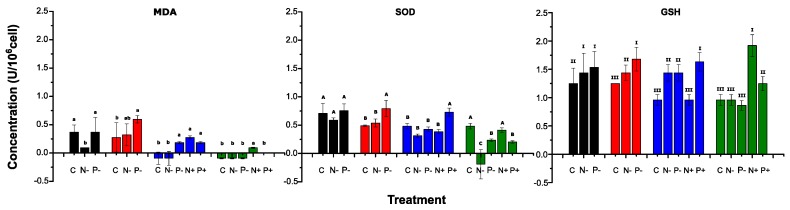
The concentration of Malondialdehyde (MDA), Glutathione (GSH) and Superoxide dismutase (SOD) of *M. aeruginosa* at each treatment. Different colors represent sampling days: Day 3 (black), Day 5 (red), Day 7 (blue) and Day 9 (green). Same letters indicate observations that are statistically indistinguishable (ANOVA, *p* < 0.05).

The MDA, SOD and GSH contents of *Microcystis* cells cultured across all the conditions have been shown in Figure 4. All data were normalized to cell density determined by absorbance at 650 nm. On Day 3, the concentration of MDA (per 10^6^ cells) in N-deficient group was lower than the control and P-deficient cultures (*p* < 0.05), but the content increased significantly on Day 5. No significant differences were seen in the concentration of SOD (per 10^6^ cells) within the treatments on Day 3, while the concentration was significantly higher in P-deficient group on Day 5. Additionally, the GSH contents (per 10^6^ cells) in nutrient deficient treatments were higher than the control (Day 3 and Day 5), with the highest content reaching about 1.75 U/10^6^ cells.

The contents of MDA, SOD and GSH changed significantly after nutrients were reintroduced. Specifically, the MDA content declined significantly in most cultures from Day 7, and those in N-deficient cultures even declined to 0. No significant differences were found in SOD contents among treatments except the P-added group on Day 7, while those in control and N-added treatments were significantly higher than the others on Day 9. The GSH content possessed the highest values in P- (Day 7) and N-added treatments (Day 9), respectively, while the concentration declined in the nutrient deficient groups significantly.

A Pearson’s correlation analysis of the correlations between MDA and enzyme activities at all treatments was displayed in Table 1. Specifically, a strong correlation was found between the contents of MDA and SOD, with *p* value of 0.002 and Pearson’s correlation coefficient of 0.7261. A moderate correlation was seen between MDA and GSH, with Pearson’s correlation coefficient of 0.5363, but the correlation was still significant (*p* < 0.05).

## 3. Discussion

There have been debates for years about the correlation between nutrient loading and harmful algae blooms and toxin productions. In this paper, we addressed the transcriptional responses of toxin formation gene and key photosynthesis genes to different nutrient loading treatments in *Microcystis aeruginosa* FACHB-905 using RT-qPCR. Our observations revealed that nutrient-deficient conditions favored the transcription of *mcy* gene and N seems more likely than P as a limiting factor in toxin production of *Microsystis.* Moreover, *Microcystis* cells presented high cell density and photosynthetic activities in P-deficient conditions, indicated by the high transcription levels of key photosynthesis genes, while cells in N-deficient conditions turned to show an adaptive regulation after a few day incubation and a long term survival eventually. The increase of antioxidant enzyme contents under an excess of ROS caused by nutrient stress suggested the ability of *Microcystis* cells in the regulation of redox system balance under stress. We consider these results in terms of the transcriptional and physiological response of *M. aeruginosa* to nutrient loading and within the context of providing more insight into naturally occurring *Microcystis* blooms and toxin regulation.

Previous reports have shown conflicting observations on the regulation of MC by the availability of N. An overall increase of MC production was seen with an increasing N availability in *M. aeruginosa* [25], while others demonstrated an increase of MC production under N-deficient conditions [26,27]. The fact that the global nitrogen regulator NtcA binding site locates in the promoter of *mcy* genes indicates the transcription of *mcy* gene cluster may itself be under direct control of N availability [23,26]. Our results are generally in accord with the hypothesis that low N concentration results in a high transcription of toxin genes, confirmed by a significant increase of *mcyB* transcripts in N-deficient condition and a rapid decrease after N was added (Figure 2). Lending support to this claim, Ginn et al. [26] reported that transcription of *mcyB* increased 14-fold in N-limited relative to N-replete treatments. Regarding P, although there have been some studies on the effect of P availability on the MC production, no conclusive results were found so far. Eldridge et al. [32] reported significant correlations between MC concentrations with total phosphorus (TP) and dissolved inorganic phosphorus (DIP) concentrations in sediment. A steady increase of *mcyD* transcripts was detected in deficiency of P with no significant changes under excess phosphate [13]. In this study, 3-fold of *mcyB* transcripts was found in P-deficient compared to the control. Although the MC content was not measured in this study, the significant high transcription of *mcyB* in nutrient-deficient treatment suggests that low nutrient concentration is potentially responsible for MC occurrence, and N is more likely than P to eventually regulate the toxin production. The addition of N would be the factor limiting of toxin production, whereas a N deficiency would allow production to continue in *Microcystis*.

Photosynthesis is tightly linked to whole cyanobacterial physiology by reciprocal controls [33]. Our results showed significant low transcription of *psaB*, *psbD* and *rbcL* in N-deficient condition, indicating the photosynthesis process was inhibited at the first three days (Figure 2). This was in consistence with Harke’s report [34], in which case, low N (75 µM nitrate) resulted in significant decrease of transcript abundance of *rbcL* and *rbcS*. These two genes encode the large (RubisCo) and small subunits of ribulose biphosphate carboxylase in photosynthesis respectively. Besides, a significant lower photosynthetic efficiency and decrease of many genes transcripts relating to photosynthesis were also observed in N-limited cultures in *M. aeruginosa* LE-3 [34]. In contrast, metabolite profiles for carbon fixation was reported to be enhanced under N starvation, and photosynthetic efficiency of PSII was significantly enhanced in the N-deficient cultures compared to the control [12]. Significantly large number of transcripts of *rbcL* and *psbD* were found in N-deficient on Day 9 in this study (Figure 2), which might suggest that the transcription profiles of key photosynthesis genes in N-limited condition varied with the culture period. Under N-limited conditions, glycogen granules could be accumulated in cyanobacteria cells [35], which act as an energy and carbon reservoir for a prolonged period of N limitation and long-term survival [12,36]. In light of this, there is still a possibility for *Microcystis* blooms under nutrient limitation.

Under P deficiency, *Microcystis* cultures could display high rate of alkaline phosphatase activity (APA) to enhance the P availability [34,37] and exhibit nearly normal growth for at least seven days [12]. Four genes (*phoX*, *sphX* and two *pstS* genes) correlated with P acquisition and transport have been observed to be largely increase of transcript abundance in P-limited condition [34]. These coincide with the observation of high transcription levels of photosynthesis genes in P-deficient condition in our study (Figure 3). Such limited condition of P-loading might give an ecological superiority for *M. aeruginosa* over other phytoplankton competitors in freshwater ecosystems. It is interesting to note that the expressions of *psaB* and *psbD* significantly declined after P was resupplied (Figure 3). Plenty of evidence has been reported that *Microcystis* was capable of phosphate uptake to form polyphosphate bodies under phosphate-replete conditions [38,39]. The synthesis of polyphosphate needs one ATP for extending P-P bond [40], which competes for energy with the photosynthesis of *Microcystis* and transcripts abundance of photosynthesis genes are likely to decline. Recently, RubisCo has been suggested to be the main binding target for MC under oxidative stress conditions [41]. Moreover, *rbcL* was reported to be repressed by NtcA binding as well, though it is not involved in N assimilation [42]. Nevertheless, no evident relationship between the *mcy* and *rbcL* gene expression has been found in this study.

It is well known that the free radicals content (O_2_^−^, H_2_O_2_, OH^−^ etc.) in phototrophs will increase under environmental stress[30,31], destroying the physicochemical properties of cell membranes [43] and disrupt the physiological activity of *Microcystis* consequently. The activity pattern of different antioxidant enzymes exhibited specific manners in different nutrient conditions. MDA is the primary product of lipid peroxidation, which reflects the degree of damage of *Microcystis* cells [44]. Contrary to our expectations, the MDA concentration (per 10^6^ cells) in N-deficient cultures were significantly lower than the control on Day 3 (Figure 4). This might be due to N-deficient conditions causing the response to intracellular ROS on GSH level, and that ameliorated the effects of MDA, which was generated at lower extent (Figure 4). This might also suggest that N limitation did not destroy the integrity of cell membrane, giving more evidences for the hypothesis that *Microcystis* cells could survive in a prolonged period of N-deficient condition. Many studies confirmed that most antioxidant enzymes activities enhance under stress conditions, like SOD, GSH, peroxidase (POD) and catalase (CAT) [45,46]. SOD and GSH act as catalysts in the reaction of O_2_^−^(ROS), converting it into the relative low cytotoxic H_2_O_2_ [47,48]. CAT can convert H_2_O_2_ into H_2_O and O_2_, defending against the oxidative damage caused by H_2_O_2_ [46]. Although the activity of CAT was not evaluated in this study, according to the measurements of SOD and GSH, we suspect the activity of CAT might present a similar trend. Significantly high contents of SOD and GSH were found in P-deficient cultures in the first five days (Figure 4), indicating that the antioxidant system was initiated to remove the excess free radical caused by the nutrient stress, but the enzymes consumed on Day 9. After the resupply of nutrients, the SOD and GSH contents increased significantly in nutrient-added treatments compared to nutrient-deficient cultures, which reflected the continuous free radical scavenging ability of cells. A strong correlation was seen between the MDA and enzyme activities (Table 1), indicating the robust effort of rebalance of the oxidation and antioxidant defense system in *Microcystis* cells.

It is also important to address the limitations of this study. The physico-chemical conditions were not measured in these incubated cultures, like the N and P concentrations at each time point. It would be interesting to explore whether nutrient-deficient cultures actually faced increased P or N concentration due to the partial cell lysis, which might give more indications on the physiological response of *Microcystis* examined in the current study. Additionally, the measurement of MC concentration and congeners should be involved, as well as the transcription of other toxin genes, to provide more insight into the regulation of toxin formation under different nutrient conditions.

The transcription profile of target genes associated with toxin formation and photosynthesis in *Microcystis aeruginosa* FACHB-905 grown in nutrient deficient and replete is an important step toward providing a broader knowledge of this toxic, bloom-forming cyanobacterium, providing proxies to ascertain cell physiology in situ as well as new insight in ongoing efforts to manage harmful algae bloom events. Future proteomic studies will help to clarify transcriptional profiles observed in this work, along with transcriptomic analyses for a comprehensive understanding of *Microcystis* physiology and MC synthesis.

## 4. Materials and Methods

### 4.1. Strain and Culture Conditions

*Microcystis aeruginosa* (FACHB-905) cultures were grown in standard BG11 medium [49] adjusted to pH 8.5. A factorial design was used to investigate the effects of N and P on the target gene expression and antioxidant enzyme activities in *M. aeruginosa.* Cells in exponential growth phase were collected by centrifugation at 6000 *g* for 10 min, and the pelleted cells were washed three times with N- and P-free BG11 medium prior to the onset of the experiments, respectively. Subsequently, cultures were inoculated in N- and P-free medium at initial cell densities of ~3.87 × 10^6^ /mL under a 12:12 light/dark cycle at 25 °C under the illumination of 40 μmol photons m^−2^ s^−1^. After a five-day treatment, NaNO_3_ (17.60 mM-N) and K_2_HPO_4_ (0.03 mM-P) were supplied as the N and P sources for half of the N- and P-deficient treatments, respectively. The concentrations were in accord with the N and P in BG11 growth medium. The other half of the cells were continually grown in N- and P-free medium until the end of sampling. Cells grown in standard BG11 medium were considered as the control. For each treatment, three independent biological replicates were established. Cell abundance was measured daily according to the previous method [8]. Samples in exponential growth phase were collected twice for RNA extraction (Day 5 and Day 9) and every other day for *M. aeruginosa* cells enzyme activity investigation.

### 4.2. RNA Extraction and Transcript Analysis

*M. aeruginosa* cells were suspended in Trizol lysis buffer (Total RNA Extractor, Sangon Biotech, Shanghai, China) for RNA extraction. Two extractions with chloroform were followed by two extractions with isopropyl alcohol. RNA was then ethanol precipitated using standard methods [50] and resuspended in RNase-free water. Contaminating genomic DNA was removed with a Turbo DNA-free kit (Ambion, Carlsbad, CA, USA). Samples were considered DNA free if no bands were visible in an agarose gel after 30 cycles of PCR amplification using 27F and 1522R primers [51]. RNA was further quantified by NanoDrop spectrophotometer and the quality and concentration for a selection of samples was verified using Agilent 2100 Bioanalyzer. A total amount of 500 ng RNA was used for the real-time reverse transcription quantitative polymerase chain reaction (RT-qPCR). The cDNA was generated using the AMV First Strand cDNA Synthesis Kit (Sangon Biotech, Shanghai, China) following the manufacturer’s protocol. cDNA samples were stored at −20 °C before running RT-qPCR.

### 4.3. Relative RT-qPCR Assay

The primers of target genes were designed using the Primer Premier 5.0 for real-time RT-qPCR (Table 2). *16S* rRNA was used as the housekeeping gene. Copy number of target genes for each sample was quantified by RT-qPCR using a ABI Stepone plus PCR system (Bio-Rad, Hercules, CA, USA).

Reaction mixtures were formulated using SybrGreen qPCR Master Mix (Bio Basic Inc., Toronto, ON, Canada). Thermal cycling conditions were: 95 °C for 3 min, 40 cycles of 3-step amplification of 7 s at 95 °C, 10 s at 57 °C and 15 s at 72 °C. Melting curves were examined to detect potential non-specific amplification. Amplification efficiency of each primer set was calculated; amplification efficiency between 90% and 110% was considered acceptable. All qPCR reactions were conducted in triplicate and the experiments were repeated at least twice. The transcript levels of each target gene were normalized by *16S* rRNA transcripts and then calculated relative to control using the 2^−ΔΔCt^. According to Plaffl [53], the ΔΔCt value of each treatment was calculated based on the following formula:

(1) $$\Delta \Delta \mathrm{Ct}={\left(C_{t}\mathrm{target}\;\mathrm{genes}-C_{t}16S\right)}_{test}-{\left(C_{t}\mathrm{target}\;\mathrm{genes}-C_{t}16S\right)}_{control}$$

### 4.4. Enzyme Extraction and Assays

*M. aeruginosa* cultures of each treatment (60 mL) were collected by a Whatman GF/C filter (0.22-μm nominal pore-size) for enzyme investigation. Liquid nitrogen grinding method was used for sample extraction and 6 mL of phosphate buffer saline (PBS, 0.05 M, pH 7.8) was used for each sample [54,55]. After centrifugation at 6000 *g* for 15 min, the supernatant was assayed for enzyme activity using a spectrophotometer (UV-2100, UNICO, Shanghai, China). Commercially available kits (MDA assay kit, T-SOD assay kit, and GSH assay kit; Nanjing Jiancheng Bioengineering Institute, Nanjing, Jiangsu Province, China) were used to determine the lipid peroxidation and antioxidant enzyme concentrations. Specifically, the thiobarbituric acid [56], xanthine oxidase [57] and 5,5′-dithiobis-2-nitrobenzoic acid [58] were used to form the colored complex of MDA, SOD and GSH respectively, and spectrophotometric analysis were used to determine the absorbance of the complex at 532, 550 and 420 nm, respectively. The concentrations were then calculated according to the formulas described by the supplier’s instructions. The MDA and enzyme concentrations were normalized to cell number before statistical analysis.

### 4.5. Statistical Analysis

All experiments were performed with three independent replicates and the data were reported as the mean ± standard deviation. One-way analysis of variance (ANOVA) was carried out to test the differences in the data of cell number, transcripts and enzyme concentrations (*p* < 0.05) between treatments using the Origin 8.0 (OriginLab, Northampton, MA, USA). Post-hoc comparisons of significant impacts were conducted with Tukey’s multiple comparison tests. T-test were conducted to examine the significant differences in transcripts between nutrient-deficient and nutrient-added treatments. A Pearson’s correlation analysis was conducted to determine the correlations between MDA and enzyme activities (*p* < 0.05).

## Figures and Tables

**Table 1:** Pearson’s correlation analysis between MDA and SOD, GSH concentrations at all treatments.

Pearson’s Correlation Analysis	*p* Value	Pearson’s R	Adj. R-Square
MDA vs. SOD	0.002	0.7261	0.4908
MDA vs. GSH	0.039	0.5363	0.2328

**Table 2:** Primers for RT-qPCR in this study

Primer	Nucleotide Sequence (5′ to 3′)	Amplification Size (bp)	Reference
			
*16S* F	GGACGGGTGAGTAACGCGTA	74	[52]
*16S* R	CCCATTGCGGAAAATTCCCC		
*mcyB* F	CCTACCGAGCGCTTGGG	77	This study
*mcyB* R	GAAAATCCCCAAAGATTCCTGAGT		
*psaB* F	CGGTGACTGGGGTGTGTATG	119	This study
*psaB* R	ACTCGGTTTGGGGATGGA		
*psbD* F	TCTTCGGCATCGCTTTCTC	90	This study
*psbD* R	CACCCACAGCACTCATCCA		
*rbcL* F	CGTTTCCCCGTCGCTTT	122	This study
*rbcL* R	CCGAGTTTGGGTTTGATGGT		
